# Impact of gender and professional education on attitudes towards financial incentives for organ donation: results of a survey among 755 students of medicine and economics in Germany

**DOI:** 10.1186/1472-6939-15-56

**Published:** 2014-07-05

**Authors:** Julia Inthorn, Sabine Wöhlke, Fabian Schmidt, Silke Schicktanz

**Affiliations:** 1 Department of Medical Ethics and History of Medicine, University Medical Center Göttingen, Humboldtallee 36, 37073 Göttingen, Germany

**Keywords:** Organ donation, Financial incentives, Survey, Students, Gender

## Abstract

**Background:**

There is an ongoing expert debate with regard to financial incentives in order to increase organ supply. However, there is a lacuna of empirical studies on whether citizens would actually support financial incentives for organ donation.

**Methods:**

Between October 2008 and February 2009 a quantitative survey was conducted among German students of medicine and economics to gain insights into their point of view regarding living and deceased organ donation and different forms of commercialization (n = 755).

**Results:**

The average (passive) willingness to donate is 63.5% among medical students and 50.0% among students of economics (p = 0.001), while only 24.1% of the respondents were actually holding an organ donor card. 11.3% of students of economics had signed a donor card, however, the number is significantly higher among students of medicine (31.9%, p < 0.001). Women held donor cards significantly more often (28.6%) than men (19.4%, p = 0.004). The majority of students were against direct payments as incentives for deceased and living donations. Nevertheless, 37.5% of the respondents support the idea that the funeral expenses of deceased organ donors should be covered. Women voted significantly less often for the coverage of expenses than men (women 31.6%, men 44.0%, p = 0.003). The number of those in favor of allowing to sell one’s organs for money (living organ donation) was highest among students of economics (p = 0.034).

**Conclusion:**

Despite a generally positive view on organ donation the respondents refuse to consent to commercialization, but are in favor of removing disincentives or are in favor of indirect models of reward.

## Background

Policy-makers and scientists in Germany are hitherto facing an unexplained contradiction. While in short surveys, the vast majority shows a passive willingness to donate, statistics do not indicate any increase in the actual supply of organs [1]. Different attempts at solving this problem such as re-organizing deceased organ donation in hospital settings have been made. However, due to the current German organ allocation scandal (which was made public by the newspaper “Süddeutsche Zeitung” in the summer of 2012) donation rates have decreased even more radically [2]. Several transplantation clinics came under investigation following allegations that doctors had falsified patients’ data or abnormally interpreted allocation rules to privilege their own patients. A commission report by the German Federal Chamber of Physicians stated that at least in the liver transplantation clinics of Göttingen, Münster, München Rechts der Isar and Leipzig breaches of law were uncovered [3]. While criminal investigations and new regulations try to rebuild public trust, the general question remains whether this will be sufficient to meet the needed number of transplantation organs in the future.

For quite some time various authors have suggested the introduction of ‘incentives’ [4-6]. The broad spectrum of suggested models ranges from direct cash payments, free market solution or indirect money saving options, here defined as ‘financial incentives’, to incentives that mirror reciprocal non-financial compensation including tokens to express social acknowledgement, bonus points in cases of being on a waiting list, or the coverage of incurred health expenses for the donor. But it is difficult to draw the line between what already counts as commercialization and what still might be seen as a balancing act of justice and fair recognition. Positions arguing for revoking the ban on organ trade [7,8] do not only have to show that ethical and legal obstacles can be overcome but also require some socio-empirical evidence suggesting that financial models will actually improve the situation.

Surveys are an important initial step to gauge relevant factors for the willingness or unwillingness to donate an organ under specific conditions. While they are seldom exact predictors of behavior, surveys can still provide very important insights into public common sense [9,10] and reveal public moralities towards organ donation as well as towards the incentive debate [11-13].

In Germany, surveys that have comprehensively tried to explore attitudes towards these issues are rare (see overview in [14]). One German study from 2001 [15] analyzed common sense opinions of 345 students of economics and medicine with a focus on organ allocation scenarios. The study found overall disagreement with the idea of a free-market based solution for organ transplantation. A comparison of valid and substantial data on attitudes of different social groups and different models is needed for the public deliberation on solutions to increase the rate of donations.

## Methods

We conducted an extensive survey among students with the aim to assess attitudes towards deceased (DOD) and living organ donation (LOD) of young people. In general, studies with young adults target an important group. During this period of life attitudes on health and values are shaped [16]. The sample was structured in a way as to compare attitudes of young adults well-informed about transplantation (medical students) with those unfamiliar with it (students of economics) and test if well-informed young adults tend to support systems of altruistic organ donation while those unfamiliar with it tend to be in favor of models of financial incentives more often. In addition to the impact caused by the professional education of the respondents, we were also interested in gender-based differences in opinions among *potential* donors. Gender differences in *actual* living donors as well as attitudes towards deceased organ donation are a well-known phenomenon across various countries, such as Germany, Switzerland, the Netherlands, Austria and the US [17-21].

The survey was conducted at the German university of Göttingen with about 23,000 students between October 2008 and February 2009. Students of medicine were chosen as a sample group (490 asked to participate, 466 participants) and this group was compared with students of economics (450 asked to participate, 289 participants) (total n = 755) (for a detailed profile of respondents see Table 1). Students were asked to participate after compulsory classes for 1^st^, 2^nd^/3^rd^ and 5^th^ year students.

**Table 1:** Profile of respondents: survey: attitudes towards organ donation

**Participants**	**Total**	**Total in %**
Medicine	466	61.7
Economics	289	38.3
Total	755	100
Female	386	52.3
Male	352	47.7
Age		
0-19 years	110	13.5
20-24 years	473	63.3
25-29 years	149	19.9
30-and above	24	3.2
Breakdown by study time	Medicine (total and (%))	Economics (total and (%))
1^st^-2^nd^ year	228 (48.8)	250 (51.2)
3^rd^ -6^th^ year	238 (86.7)	35 (13.3)

The study was conducted with healthy adults by using an anonymous questionnaire. According to the local ethics committee at the University Medical Center Göttingen, no formal approval for this kind of research is needed. All participants were informed about the aim of the study, gave their written informed consent (on a separate sheet of paper from the questionnaire) and participated on a strictly voluntary basis.

Questions on financial models are based on models already used in studies in the US by [21,22] and in the UK by [23]. In total, the questionnaire consisted of 55 sets of closed questions addressing the following topics: prior knowledge about organ transplantation and allocation, attitudes towards living and deceased donation under different conditions, models of commercialization and incentives, concepts of death and bodily identity, consent models, pro-social behavior and socio-demographic data based on [24]. We used a 6-Likert-scale for questions on attitudes, and yes/no/don’t know for questions on knowledge and for simple questions on decisions or willingness (for the full questionnaire see Additional file 1). The questionnaire was pre-tested for comprehensibility and factor analysis was used to explore validity. The data was analyzed using data processing software SPSS (version 21.0, 2012). The analysis is based on frequency analysis and calculation of means for Likert-scales. Differences between groups (field of study, gender) were studied by applying Chi-square tests using a significance level of p = 0.05.

## Results

### Sample and response rate

The sample included 466 students of medicine (response rate: 95.1%) and 289 students of economics (response rate: 64.2%). In total n = 755 students in their first to sixth year of study participated. The overall response rate was 80.3% with a significant difference in response rates between students of medicine and economics (p < 0.001) (for a detailed profile of respondents see Table 1).

### Willingness for DOD

**Figure 1:**
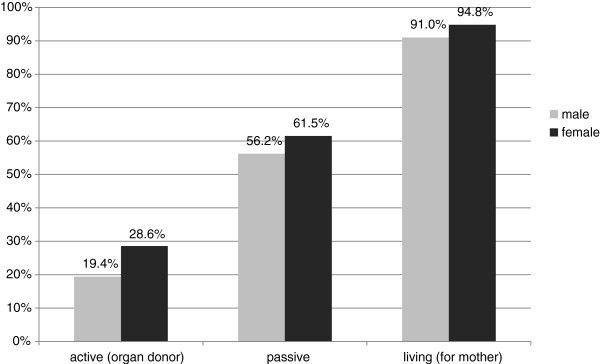
Willingness for altruistic organ donation: gender differences.

In questions on altruistic donation after death, we distinguished between active and passive willingness. Passive willingness means that a person is principally willing to donate an organ, while carrying a donor card means active willingness to donate.^a^ The passive willingness for deceased organ donation was 58.4% among all participating students, while 33.2% were undecided. Interestingly, more medical students (63.5%) than students of economics (50.0%) were principally willing to donate an organ (p = 0.001).The data set, however, shows that only a small number of students who were passively willing to DOD actually carried a donor card (see Figure 1).

The academic field of study can be seen as a significant influence. While 11.3% of students of economics have signed a donor card, the number is significantly higher among students of medicine (31.9%, p < 0.001).

The most frequent explanation for not having donor card is based on undecidedness (32.8%) and ‘not having thought about the issue’ (25.6%). Of all the respondents who were not carrying a donor card, 45.9% would still be willing to DOD. 10.8% would explicitly reject donating organs and 43.3% are undecided.

### Willingness for LOD

Across all students the willingness to agree to LOD is much higher than to DOD (see Figure 1).

In the case of a sick partner 80.3% of men (men → women) and 85.6% of women (women → men) would be willing to help their partner with a living donation. The willingness to donate a living organ to one’s own child is equally high across both groups (to daughter: women: 85.9%, men: 85.1%; to son: women 82.5%, men: 78.4%). The question on possible motivations for LOD showed a clear ranking of reasons (ranking each motivation from 1 = total approval – 6 = total disapproval). The primary motivation was *love* (mean 1.32). The second most important motivation was *responsibility towards the family* (mean 2.24). *Moral duty* as a motivation is seen ambivalently (mean 3.24). Other motivations such as to *live up to the expectation of the family*, *gaining social approval* or *financial compensation* gained low or no consent (mean ≥ 4.80). The most frequently cited reason against LOD was fear of medical complications (women: 74.9%; men: 67.9%) (p = 0.029 sig.), followed by the statement that this constitutes an invasion of bodily integrity (women: 47.9%; men: 42.9%) (n.sig.). 11.7% of women and 15.6% of men were of the opinion that this also invades one’s ‘psychological integrity’ (n.sig.).

Although anonymous LOD is prohibited in Germany, 44.3% of all respondents were in favor of it (students of economics: 47.1%; students of medicine: 42.7%) (p = 0.036 sig.). Students agreed that LOD should also be allowed between individuals who are not related or friends, but are ‘on bowing terms’. (students of economics: 75.3%; students of medicine: 63.9%) (p < 0.001 sig.).

### Attitudes towards financial and non-financial incentives for DOD and LOD

#### Attitudes towards incentives for deceased organ donation

Students were asked about different models of commercialization and compensation for organ donation. Here the comparison between students of medicine and economics was most relevant. The two groups differ significantly in most of their answers on different economic models.

Although both groups tend to reject financial models, the number of voices in favor of financial incentives was always higher among students of economics.

All in all, respondents are rather skeptical about ideas involving direct payments or financial incentives for DOD (see Table 2). Yet, different options show a differing extent of rejection. 72.4% of students of medicine, but only 52.7% of students of economics reject the option of paying a lump sum to the deceased person’s family as a token of appreciation (p < 0.001). The majority of respondents reject any and all financial advantages for potential donors during their lifetime such as tax benefits (rejected by 70.8%) or cash payments (rejected by 73.4%).

**Table 2:** Positive attitude towards models of financial and non-financial incentives for DOD

**In %**	**Total**	**Men**	**Women**	**Sig.**	**Medicine**	**Economics**	**Sig.**
Recipient’s health insurance makes a donation	18.8	21.6	15.8	n.s.	16.0	23.5	p = 0.015
The insurer helps the deceased donor’s family by covering funeral expenses	37.8	44.0	31.6	p = 0.003	37.8	37.7	n.s.
The insurer pays the bereaved as a token of appreciation	14.5	17.4	10.7	p = 0.02	9.8	22.3	p = 0.000
Those who fill in a donor card get tax benefits	11.7	14.5	9.1	n.s.	11.7	11.9	n.s.
Donor card holders receive one-off payment	7.8	10.4	4.9	p = 0.014	5.9	11.1	p = 0.013
Donor card holders get bonus points on organ waiting list	46.5	50.4	42.9	p = 0.043	41.1	55.5	p = 0.000

Options to answer were yes/no/don’t know, here only positive answers are shown.

Covering the deceased donor’s funeral expenses is assessed as appropriate by 37.7% of students of economics and 37.8% of students of medicine (see Table 2), but one fifth (students of economics: 23.9%; students of medicine: 20.0%) are undecided. Students were even more positive about awarding bonus points in a future case of one being on a recipient’s waiting list (students of economics: 55.5%; students of medicine: 41.1%) (p < 0.001 sig.) (see Table 2).

The relatively high number of students who are undecided on the topic of financial incentives for deceased donation is noteworthy. A greater number of students are undecided than in favor of an option.

Overall, women tend to be more critical towards models of most incentives for DOD; for 4 out of 6 models, the differences are significant (see Table 2).

#### Attitudes towards incentives for living organ donation

With regard to LOD, only 5.0% of students of medicine and 9.1% of students of economics were in favor of allowing to sell one’s organs for money (p = 0.034 sig.). Women were more likely to vote against this option than men (p = 0.005 sig.). The majority of respondents (73.1%) held that a living organ donor should receive cheaper or free follow-up treatment, while only a minority thought it appropriate that a living donor should receive free life insurance from the state (8.9%) (see Table 3). Overall, models which can be seen as removing disincentives such as compensation for health and surgery related costs or models of reciprocity (organ donors benefit in case they need an organ themselves) gain much higher approval than models that can be described as sheer monetary ‘incentives’ (see Table 3).

**Table 3:** Positive attitudes towards financial and non-financial incentives for LOD

**In %**	**Total**	**Men**	**Women**	**Sig.**	**Medicine**	**Economics**	**Sig.**
Get tax benefits	12.6	18.2	7.9	p = 0.000	12.0	13.4	n.s
Financial compensation for loss of earnings	71.3	73.3	69.6	n.s.	69.1	75.0	n.s.
Free accident insurance	22.4	28.0	16.9	p = 0.000	20.6	25.4	n.s.
Free pension and accident insurance	13.8	16.1	11.0	p = 0.016	13.8	13.7	n.s.
Be allowed to sell their organ for money	6.5	9.6	3.7	p = 0.005	5.0	9.1	p = 0.034
Get a reduction in health care insurance fees	44.5	46.5	42.7	n.s	41.6	49.4	n.s
Get private health insurance	12.4	13.4	11.6	n.s	12.1	13.0	n.s.
Receive subsidized or free follow-up treatment	73.1	69.4	76.7	n.s	70.3	77.6	n.s.
Receive free life insurance from the state	8.9	11.2	6.8	n.s.	7.5	11.2	n.s.
Bonus points for receiving an organ in case of an own illness	54.1	54.9	54.0	n.s	49.7	61.9	p = 0.004

Options to answer were yes/no/don’t know, here only positive answers are shown.

**Figure 2:**
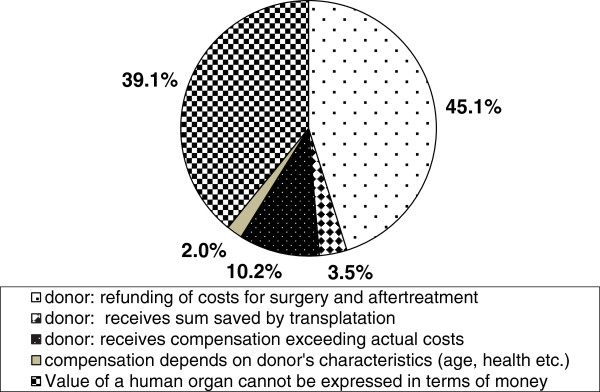
Comparison of models of financial and non-financial incentives for LOD.

**Figure 3:**
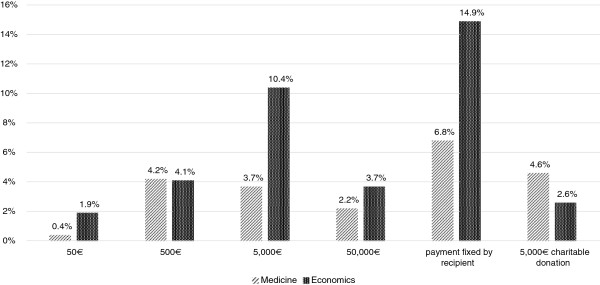
Subject differences in the acceptance of a fee for living organ donation.

All questions on commercial models indicate a tendency for gender differences. Women reject financial compensation more readily than men. Thus, the approval on tax benefits for living donors shows a significant difference between men (18.2%) and women (7.9%) (p < 0.001 sig.). The vast majority of medical students are negative about this possibility (women: 79.1%; men: 66.8%) (p < 0.001 sig.). Considering the option of a free accident insurance for a living organ donor, more men (students of medicine: 25.8%; students of economics: 31.3%) than women (students of medicine: 16.0%; students of economics: 18.6%) are in favor of such a scheme (total gender difference p < 0.001 sig.) (see Table 3).When asked about their general attitude towards financial compensation for LOD, respondents were in favor of compensation of costs for surgery and medical expenses directly associated with the donation (which is normally the case in industrialized countries) while there was little support for the idea of financial, cash rewards (see Figure 2).When having to choose between different models of compensation, it is still noteworthy that less than half of the students (45.2% of students of medicine, 44.9% of students of economics) think that donors should be compensated for those health expenses (see Figure 2). However, more students of economics are of the opinion that financial compensation should exceed the costs incurred than medical students (students of economics: 15.9% vs. students of medicine: 7.1%). Also, students of medicine and economics differ on the belief that the value of an organ cannot be expressed in terms of money (students of economics: 32.7% vs. students of medicine: 42.7%, p = 0.002 sig.).When asked about the adequate sum of compensation 72.3% of the respondents totally rejected the idea of financial compensation no matter whether the remuneration for living organ donation was set at 50€, 500€, 5,000€ or 50,000€. Acceptance was always below 5% (total data set). Only the answer of students of economics in favor of a one-off payment worth 5,000€ deviated from this pattern with 10.4% agreeing to this model (see Figure 3).

## Discussion

Social-empirical studies, whether quantitative or qualitative, cannot and should not replace ethical, legal, public and political deliberation on controversies in modern democracies. However, they have an important function to inform experts and policy-makers about the validity of their assumption on what the public approves or disapproves of.

The survey is neither representative of the German population nor of young people in general as our respondents are highly educated and their fields of study are related to the investigated topics. However, the comparison between students of medicine and economics provides valuable information on the impact of knowledge about organ donation and attitudes towards LOD and DOD. Our data collection was carried out in Göttingen before the local organ allocation scandal became public and shows strong commonalities with other representative survey among German citizens before 2012 (see below). This strengthens our assumption that our sample does not differ strongly from other sections of the German population and therefore provides insights into general public attitudes towards organ donation and the influence of gender.

For DOD we found that overall passive willingness to donate is much higher than active willingness, but still passive willingness was expressed by little less than 2/3 of all respondents. Medical knowledge and thus knowledge about organ donation has an important impact on the (active and passive) willingness to DOD. This is coherent with results from intervention studies in Germany [25,26]. Figueroa et al. [19] found similar results about the relevance of information for the willingness to DOD in the Netherlands (see also [27]). Notably, the ratio between active and passive willingness to donate matches very well with other German surveys [1,28,29]. Also, a more comprehensive representative study with 1,000 German citizens [30] on attitudes towards presumed consent and market models for organ donation found that 59% were passively willing to donate, but only 13% hold a donor card. This corresponds to practice: Only 2/3 of family members agree to DOD (on behalf of the patient’s anticipated wish), while 1/3 disagree with it [31].

With regard to LOD, the willingness to donate a living kidney to a known and beloved person is much higher than to an unknown person in the context of deceased donation (similar: [28,30]). This is also true for medical students among which good medical and scientific knowledge of organ transplantation, including the medical risks, can be assumed. The German findings concerning LOD are remarkably higher than findings of other European studies (40% in the Netherlands [32] and 80% in the UK [33]). The high willingness contrasts the present legal situation (LOD makes up less than 1/3 of all donations [34]) and recent political measures in Germany. The German law continues to consider LOD as a second-rate option compared to DOD. It is only an option if no organ from a DOD is available. Thus, although there seems to be a high potential for LOD, this option cannot be further promoted by doctors.

With regard to attitudes towards different models of incentives, multiple gender differences can be observed. Since several international surveys do not report any gender differences, this finding is remarkable [32,35]. However, our findings are congruent with another German representative survey [30]. Decker et al. concluded that men were significantly more positive about the idea of financial incentives, including cash payments for organ donation, framing the result as ‘sex sells cells’. Likewise, a US internet survey [4] and a Scottish study [36] established that women rather disapprove of market models for LOD and DOD. Cultural and gender differences on opinion levels (surveys) are mirrored on the behavioral level (donation practice) (see [17]) and convincingly indicate a socially gendered discourse and practice. However, a natural difference in altruistic behavior between women and men, as discussed by some authors [37,38], is less plausible.

Finally, our most interesting results concern the different views of our respondents with regard to different models of reward, compensation and financial incentives. The systematic pattern we found indicates that the majority of respondents clearly prefers models that cannot be classified as sheer ‘incentives’ and monetary motivation, but as a kind of fair compensation for health and surgery-related (e.g. such as after treatment) costs which can also be interpreted as removing disincentives [39].

Hence, we see a very clear-cut picture in LOD: *altruistic* donation does not imply, for the majority, that the donor gives his/her organ(s) *and* additionally has to cover surgery-related costs. Decker et al. [40] found that the clear majority is against direct payments by the recipient, while over half of the respondents supported the idea that the expenses (not defined) are covered by the government/health insurances. Likewise, the telephone survey by Boulware et al. [21] with US citizens found even higher support for reimbursing medical costs (91%) and paying sick leave (84%) in LOD.

In DOD, the model of compensation for funeral costs is approved by over 1/3 of our respondents. However, questions regarding this topic seem to produce very heterogeneous results. In two US studies the approval ranges from 9% [21] to 81% [22]. In Decker et al. [30] 51% of men and 45% of women approved of the coverage of funeral costs, which is in line with Haddow [36] finding 44% positive votes.

The idea of being given priority on the waiting list for being a donor card holder or as a living donor, as realized by only very few countries such as Israel [41], gained close to half of all votes. Likewise, Boulware et al. [21] found an approval of this idea by the majority (59%).

Overall support is given to models that award compensation for health costs for the donor by indirect acknowledgements or reciprocity (such as the bonus on a waiting list) [42]. Free market models and sheer cash incentives find very low support. This can be interpreted as a common sense of fairness, reciprocity as incentive or as removing disincentives, but does not support ideas that bodily self-ownership, free market or cash as incentives will solve the problem of organ shortage.

## Conclusion

Our findings concerning the common sense for more compensation and reward models contrast with the classic dichotomy between altruism on the one hand and commodification, by paid donations, on the other.

Given the gender differences observed in Germany, we recommend that policies and rhetoric for organ donation, health care and social security should be critically investigated for any possible gender biased agendas.

While it is ethically and legally problematic to base politics on majorities in surveys, surveys are very helpful to understand the common sense. Continuous low support for financial incentives as well as moderate support for organ donation does not support recent proposals for paid donation. However, transparent public debates on indirect and health-care related compensation and on safeguarding the donors can be seen as timely and much more appropriate than insisting on altruism.

## Endnote

^a^According to German law deceased organ donation is only allowed with persons who gave their consent during lifetime. This is usually done by filling in a donor card.

## Abbreviations

LOD: Living organ donation; DOD: Deceased organ donation.

## Competing interests

The authors declare that there are no competing interests.

## Authors’ contributions

JI did the data analysis and participated in writing of the paper; SW participated in writing of the paper and in research design; FS did the acquisition of data; SiS participated in research design and writing of the paper. All authors revised the manuscript critically, all authors read and approved the final manuscript.

## Pre-publication history

The pre-publication history for this paper can be accessed here:

http://www.biomedcentral.com/1472-6939/15/56/prepub

## Supplementary Material

Additional file 1 Questionnaire Organ Donation. Click here for file
