# Supplementary material for: Impact of gender and professional education on attitudes towards financial incentives for organ donation: results of a survey among 755 students of medicine and economics in Germany
Source: BMC Med Ethics. 2014 Jul 5;15:56. doi: 10.1186/1472-6939-15-56 (PMC4107572; doi:10.1186/1472-6939-15-56)
Supplement: Additional file 1 — Questionnaire Organ Donation. [file 1472-6939-15-56-S1.docx]

**Questionnaire Organ Donation**

**Before you start to fill in the questionnaire, we would like to briefly explain what we mean by living organ donation and deceased organ donation as we assume that not everyone will be familiar with the terms:** When people make their organs available for donation after death, this is called deceased organ donation. Organs of deceased can only be donated if so-called brain death of the donor has been determined. In 2007, 4885 organs were donated postmortem (2340 kidneys, 1088 livers, 394 hearts, 284 lungs, and 139 pancreas).

Since the German organ transplantation act has been passed in 1997, living organ donation is another possibility available in addition to deceased organ donation. In living donation, especially kidneys and part of the liver can be transplanted. According to German law and in contrast to deceased organ donation, living donation is not anonymous. Living donation can only take place between first and second degree relatives and very close friends. Between 1990 and 2005 the number of living transplantations rose to 522 annually.

**First we would like to know some facts about your degree course:**

For students of medicine:

Which semester are you in?

|  |  |
| --- | --- |

For students of economics / business studies:

Which degree course are you currently enrolled in?

| BSc Business | BSc Economics |
| --- | --- |
| MSc Finance, Accounting, and Taxes | MSc Marketing and Channel Management |
| MSc Management | MSc International Economics |

Which semester are you in?

Did you obtain your university entrance qualifications in Germany?

If not, where?

**We will start by asking you about your prior experiences concerning organ transplantation.**

Do you carry a donor card?

| Yes | No |
| --- | --- |

If not: which of these reasons would best explain why you do not carry a donor card:

*Multiple answers are possible!*

| I haven’t yet thought about the issue. | I haven’t had the opportunity to fill in a donor card. |
| --- | --- |
| I’m undecided whether I want to fill in a donor card. | Personally, I reject carrying a donor card. |
| I do not carry a donor card because I’m afraid that physicians might be more interested in patients’ organs than in saving their life. | I do not carry a donor card because I’m afraid that organs might be removed before the patient has died. |
|  | |

Have you already thought about the topic of organ donation?

| Yes, I have dealt with this topic. | I have only considered it a little. |
| --- | --- |
| No, I have never considered it |  |
|  |  |

Have you ever heard about living organ donation?

| Yes | No |
| --- | --- |
|  |  |

Is there anyone in your circle of family and friends who has donated an organ?

| Yes | No |
| --- | --- |
|  |  |

Is there anyone in your circle of family and friends who has received an organ?

| Yes | No |
| --- | --- |
|  |  |

Is there anyone in your circle of family and friends who is suffering from a chronic organ disease?

| Yes | No |
| --- | --- |
| Don’t know |  |
|  |  |

If you have heard about living organ donation before, where did you get the information?

*Multiple answers are possible!*

| Family / relatives | Magazines / newspapers |
| --- | --- |
| Internet | Friend |
| Patients’ organization / support group | TV or radio ads |
| Leaflets | Posters |
| Physician | Books |
| Movie or TV show | Lecture |
| Don’t know | I don’t have any information |
|  |  |

With whom have you already talked about organ transplantation?

*Multiple answers are possible!*

| Mother | Wife / partner (female) / girlfriend |
| --- | --- |
| Father | Close friends |
| Brother | Physician |
| Sister | I’d rather not talk about it |
| Daughter | None |
| Son | Other: |
| Husband / partner (male) / boyfriend |  |
|  |  |

**In the following section, we would like to ask about your opinion on possible organ donation.**

Would you donate your organs after death?

| Yes | No |
| --- | --- |
| Don’t know |  |
|  |  |

Which organs would you donate postmortem (after death)?

*Multiple answers are possible!*

| Liver | Larger area of skin |
| --- | --- |
| Pancreas | Kidney |
| Hand, foot | Cornea |
| Heart | None |
| Lungs |  |
|  |  |

To remove organs after death, so-called brain death has to be determined.

Which of these statements do you agree with?

*Please tick* ***one*** *answer only!*

| When a person's brain completely stops functioning, the person is dead. | When those regions of the brain connected with personality, thinking, and speaking stop functioning, the person is dead. |
| --- | --- |
| Even if the brain is irreversibly damaged, a person is not dead as long as the other organs are still functioning. |  |
|  |  |

Living organ donation is the voluntary donation of a kidney or part of the liver. In case of illness, would you donate an organ to the persons listed below?

*Please tick one answer in* ***each*** *line!*

|  | Yes | No | Can’t say |
| --- | --- | --- | --- |
| Mother |  |  |  |
| Father |  |  |  |
| Brother |  |  |  |
| Sister |  |  |  |
| Husband / partner (male) / boyfriend |  |  |  |
| Wife / partner (female) / girlfriend |  |  |  |
| Daughter |  |  |  |
| Son |  |  |  |
| Cousin (female) |  |  |  |
| Cousin (male) |  |  |  |
| None |  |  |  |
| Others: |  |  |  |
|  |  |  |  |

If someone in your family was sick and you had to decide whether to donate an organ, whom would you ask for advice?

*Please tick one answer in* ***each*** *line!*

|  | | Fully applies |  |  |  |  | Does not apply |
| --- | --- | --- | --- | --- | --- | --- | --- |
| Spouse / partner | | 1 | 2 | 3 | 4 | 5 | 6 |
| Close family | |  |  |  |  |  |  |
| Friends | |  |  |  |  |  |  |
| Patients / relatives | |  |  |  |  |  |  |
| Physicians | |  |  |  |  |  |  |
| Pastor / minister | |  |  |  |  |  |  |
| Psychologist | |  |  |  |  |  |  |
| Social worker | |  |  |  |  |  |  |
| Caregivers | |  |  |  |  |  |  |
| Support group | |  |  |  |  |  |  |
| Other: | |  |  |  |  |  |  |
|  |  | | | | | | |

What would motivate you to donate a living organ, e.g. a kidney, to a family member?

*Please tick one answer in* ***each*** *line!*

| **I would donate an organ to a person…** | | Total approval |  |  |  |  | Total disapproval | |
| --- | --- | --- | --- | --- | --- | --- | --- | --- |
| out of love or emotional closeness. | | 1 | 2 | 3 | 4 | 5 | 6 | |
| because I feel responsible towards my family. | |  |  |  |  |  |  | |
| because of a sense of moral duty. | |  |  |  |  |  |  | |
| because I want to live up to my family’s expectations. | |  |  |  |  |  |  | |
| because I would gain social recognition. | |  |  |  |  |  |  | |
| if I received financial compensation. | |  |  |  |  |  |  | |
|  |  | | | | | | |  |

How do you think the relationship between donor and recipient would change after living organ transplantation?

*Please tick* ***one*** *option only!*

| Improve significantly | Deteriorate somewhat |
| --- | --- |
| Improve somewhat | Deteriorate significantly |
| Stay the same |  |
|  |  |

Why would you potentially decide against donating an organ?

*Multiple answers are possible!*

| It constitutes an invasion of my bodily integrity. | It constitutes an invasion of my psychological integrity. |
| --- | --- |
| Fear of medical complications. | Lack of trust in physicians / medical care. |
| Fear that the recipient’s body will reject my organ. | None of the above. |
|  |  |

**In the following section, we will ask you about how you would act if you were sick and in need of an organ.**

If you were sick and in need of an organ, who would you ask for advice on whether to decide for or against organ transplantation?

*Please tick one answer in* ***each*** *line!*

|  | | Fully applies |  |  |  |  | Does not apply |
| --- | --- | --- | --- | --- | --- | --- | --- |
|  | | 1 | 2 | 3 | 4 | 5 | 6 |
| Spouse / partner | |  |  |  |  |  |  |
| Close family | |  |  |  |  |  |  |
| Friends | |  |  |  |  |  |  |
| Patients / relatives | |  |  |  |  |  |  |
| Physicians | |  |  |  |  |  |  |
| Pastor / minister | |  |  |  |  |  |  |
| Psychologist | |  |  |  |  |  |  |
| Social worker | |  |  |  |  |  |  |
| Caregivers | |  |  |  |  |  |  |
| Support group | |  |  |  |  |  |  |
| Other: | |  |  |  |  |  |  |
|  |  | | | | | | |

Imagine you need an organ donated by a brain-dead patient to survive. How important would the following background information on the donor be in this situation?

*Please tick one answer in* ***each*** *line!*

|  | Very important |  |  |  |  | Not important at all |
| --- | --- | --- | --- | --- | --- | --- |
|  | 1 | 2 | 3 | 4 | 5 | 6 |
| Age |  |  |  |  |  |  |
| Character |  |  |  |  |  |  |
| Sex |  |  |  |  |  |  |
| Fitness |  |  |  |  |  |  |
| Smoking and drinking habits |  |  |  |  |  |  |
| Nationality |  |  |  |  |  |  |
| Religious affiliation |  |  |  |  |  |  |
| Health |  |  |  |  |  |  |
| Skin colour |  |  |  |  |  |  |
| Criminal record |  |  |  |  |  |  |
| Other: |  |  |  |  |  |  |
|  |  |  |  |  |  |  |

In the case that you were in need of an organ, which of the following organs would you accept?

*Please tick one answer in* ***each*** *line!*

|  | | Fully accept |  |  |  |  | Never accept |
| --- | --- | --- | --- | --- | --- | --- | --- |
|  | | 1 | 2 | 3 | 4 | 5 | 6 |
| Heart | |  |  |  |  |  |  |
| Liver | |  |  |  |  |  |  |
| Pancreas | |  |  |  |  |  |  |
| Lung | |  |  |  |  |  |  |
| Kidneys | |  |  |  |  |  |  |
| Cornea | |  |  |  |  |  |  |
| Face | |  |  |  |  |  |  |
| Eyes | |  |  |  |  |  |  |
| Individual limbs (e.g. a hand, arm, or leg) | |  |  |  |  |  |  |
| Genitalia | |  |  |  |  |  |  |
|  |  | | | | | | |

If you were in need of an organ donation to survive, which organ would you prefer?

*Please tick one answer in* ***each*** *line!*

|  | | Yes | No | Don’t know |
| --- | --- | --- | --- | --- |
| The organ of a brain-dead patient | |  |  |  |
| The organ of a living donor | |  |  |  |
| An organ of an animal (e.g. pig, monkey) (xenotransplant) | |  |  |  |
| An organ grown from stem cells | |  |  |  |
| An artificial organ (a machine) | |  |  |  |
| I don’t care as long as I recover | |  |  |  |
| I oppose any kind of organ transplantation | |  |  |  |
|  |  | | | |

Imagine you were ill and in need of a new organ from a living donor. Who would you accept as a living donor?

*Please tick one answer in* ***each*** *line!*

|  | Yes | No | Can’t say |
| --- | --- | --- | --- |
| Mother |  |  |  |
| Father |  |  |  |
| Brother |  |  |  |
| Sister |  |  |  |
| Husband / partner (male) / boyfriend |  |  |  |
| Wife / partner (female) / girlfriend |  |  |  |
| Daughter |  |  |  |
| Son |  |  |  |
| Cousin (female) |  |  |  |
| Cousin (male) |  |  |  |
| Other relatives |  |  |  |
| Friend (female) |  |  |  |
| Friend (male) |  |  |  |
| None |  |  |  |
| Others: |  |  |  |
|  |  |  |  |

**The following questions address so-called “xenotransplantation”. Xenotransplantation means that organs or part of an organ are transferred from an animal to a human.**

Have you ever heard of transplantation of animal organs to humans before?

| Yes | No |
| --- | --- |
| Don’t know |  |
|  |  |

Imagine you depended on an organ transplantation to survive: would you accept an animal organ transplant if the success rate of such a transplantation was similar to human transplants?

| Yes | No |
| --- | --- |
| Don’t know |  |
|  |  |

What kind of fears or anxiety would you have concerning animal organ transplants?

*Please tick one answer in* ***each*** *line!*

|  | Very concerned | |  |  |  |  | Not concerned | |
| --- | --- | --- | --- | --- | --- | --- | --- | --- |
|  | 1 | | 2 | 3 | 4 | 5 | 6 | |
| Emotional distress |  | |  |  |  |  |  | |
| Change in my personality |  | |  |  |  |  |  | |
| Religious issues |  | |  |  |  |  |  | |
| Danger of rejection / poorer functioning than a human organ |  | |  |  |  |  |  | |
| Transmission of diseases / infections |  | |  |  |  |  |  | |
| Animal welfare |  | |  |  |  |  |  | |
| Other: |  | |  |  |  |  |  | |
|  | |  | | | | | |  |

**The following questions concern your image of the human body.**

Which of the following statements on the human body would you agree with?

*Please tick one answer in* ***each*** *line!*

|  | Yes | No | Don’t know |
| --- | --- | --- | --- |
| The human body is comparable to a machine; individual parts can easily be replaced. |  |  |  |
| The human body is more than the sum of its parts; thus, individual parts cannot always just be replaced. |  |  |  |
| Certain organs determine a person’s individuality and uniqueness and should not be transplanted. |  |  |  |
| The heart is the location of the soul and should not be transplanted. |  |  |  |
|  |  |  |  |

Which of the following body parts / organs should principally be allowed for transplantation?

*Please tick one answer in* ***each*** *line!*

|  | | Yes | No | Don’t know |
| --- | --- | --- | --- | --- |
| Brain | |  |  |  |
| Genitalia | |  |  |  |
| Eyes | |  |  |  |
| Face | |  |  |  |
| Individual limbs (e.g., hand, arm, or leg) | |  |  |  |
|  |  | | | |

**Please answer a few questions discussed in public discourse and politics.**

Which statements on organ transplantation do you agree with?

*Multiple answers are possible!*

| There is a shortage of organs | There is a problem concerning allocation |
| --- | --- |
| There is a problem concerning the use of brain-dead patients as organ donors | There is a problem concerning the occurrence of changes in identity and personality after organ donation |
| Organ transplantation is well regulated |  |
|  |  |

In public debate, some argue for the extension of living organ donation to persons outside of the family. Thus, they advocate the introduction of anonymous living donation (donor and recipient do not know each other).

*Please tick one answer in* ***each*** *line!*

|  | Yes | No | Don’t know |
| --- | --- | --- | --- |
| Living donation should only be allowed between related persons. |  |  |  |
| Living donation should also be allowed between unrelated persons who are only on bowing terms. |  |  |  |
| Anonymous living donation should be introduced. |  |  |  |
|  |  |  |  |

In Germany and other European countries 2/3 of all living organs are donated by women. In your opinion what could be the reason for this?

*Please tick one answer in* ***each*** *line!*

|  | Yes | No | Don’t know |
| --- | --- | --- | --- |
| Women feel more responsible for the well-being of family members. |  |  |  |
| More men suffer from chronic diseases than women. |  |  |  |
| Women live a healthier life than men. |  |  |  |
| Men fall sick earlier than women. |  |  |  |
| Women are better at tolerating pain than men. |  |  |  |
| Men are better at accepting help than women. |  |  |  |
| Women are more caring than men. |  |  |  |
| Men will more readily admit and talk about pain than women. |  |  |  |
| Women are better positioned economically to run the risk of living donation, as they do not tend to be responsible for the social and financial security of their family in case of complications. |  |  |  |
| Men are responsible for providing for their family with social and financial security and are thus reluctant to run health risks. |  |  |  |

In Germany, organs can only be removed if donors agreed before their death or if their families expressly agreed after their death (opt-in system). In other countries, those who do not want to donate can at any time register their objection. Under this legislation, organs are removed after death if the person did not refuse during life time (opt-out system). As a third possibility, all citizens of legal age could be compelled to make a choice on organ donation (positive or negative) (mandated choice).

*Please tick* ***one*** *answer only!*

| I’m in favor of retaining the opt-in system | I’m in favor of the opt-out system |
| --- | --- |
| I’m in favor of the introduction of a mandated-choice system | I don’t know |
|  |  |

**Experts discuss whether organ shortage can be countered by commercializing organ donation. In this section, we will ask you some questions on this topic.**

In public debates on organ donation, financial incentives are regularly brought up as a measure to increase the number of organ donors. Do you think that a living organ donor should…

*Please tick one answer in* ***each*** *line!*

|  | | Yes | No | Don’t know |
| --- | --- | --- | --- | --- |
| get tax benefits | |  |  |  |
| receive financial compensation for loss of earnings | |  |  |  |
| receive free accident insurance | |  |  |  |
| receive free pension and accident insurance | |  |  |  |
| be allowed to sell their organ for money | |  |  |  |
| receive a reduction in health care insurance fees | |  |  |  |
| receive private health care insurance | |  |  |  |
| receive cheaper or free follow-up treatment | |  |  |  |
| receive free life insurance from the state | |  |  |  |
| get a bonus on the waiting list and be given preference if they themselves should need a living organ donation | |  |  |  |
|  |  | | | |

Which of the following statements on financial compensation for living donors do you agree with:

*Please tick* ***one*** *answer only!*

| Donors should receive the sum they had to pay for surgery and follow-up treatment (e.g., travel costs, medication, etc.). | Donors should receive the amount of money which the health insurer saved due to the donation and which would have otherwise followed from chronic organ failure (e.g. dialysis.). |
| --- | --- |
| Donors should receive compensation which exceeds the costs incurred. | The sum depends on the donor’s specific situation (e.g. age, health). |
| The value of a human organ cannot be expressed in terms of money. |  |
|  |  |

Some people have proposed that donors should receive a one-off payment. Do you think that a living donor should receive…

*Please tick* ***one*** *answer only!*

| A fixed payment of 50 euro | A fixed payment of 500 euro |
| --- | --- |
| A fixed payment of 5.000 euro | A fixed payment of 50.000 euro |
| A sum determined by the recipient | No payment |
| A payment of 5.000 euro to donate to a charity of the donor’s choice |  |
|  |  |

In organ transplantation, organs from brain-dead patients are used more often than living organs. Do you think that the following economic incentives for postmortem donation are acceptable?

*Please tick one answer in* ***each*** *line!*

|  | | Yes | No | Don’t know |
| --- | --- | --- | --- | --- |
| Organ recipient’s health insurance makes a donation on behalf of the deceased donor. | |  |  |  |
| The insurer helps the deceased donor’s family by covering funeral expenses. | |  |  |  |
| The insurer pays the bereaved as a token of appreciation. | |  |  |  |
| Those who fill in a donor card get tax benefits. | |  |  |  |
| Donor card holders receive one-off payment. | |  |  |  |
| Donor card holders get bonus points on organ waiting list should they be in need of an organ. | |  |  |  |
|  |  | | | |

**We will now ask you some questions about yourself and your personal circumstances.**

I am …

| Male | Female |
| --- | --- |
|  |  |

How old are you?

| Younger than 20 | 20 to 24 |
| --- | --- |
| 25 to 29 | 30 to 39 |
| 40 to 49 | 50 to 59 |
|  |  |

Do you have any children?

| Yes | No |
| --- | --- |

How many?

|  |  |
| --- | --- |

What is your marital status?

| Single | Married |
| --- | --- |
| Civil partnership | In a relationship |
| Widowed | Divorced |
| Separated |  |
|  |  |

Do you live with…

*Multiple answers are possible!*

| Your spouse | Your partner |
| --- | --- |
| Your children | Your parents |
| Flat mates | No one |
| Other: |  |
|  |  |

Please tick the characteristics with which **you** identify. If you are not living in a relationship, you can answer those questions based on previous relationships.

*Please tick one answer in* ***each*** *line!*

|  | Fully Agree | |  |  |  |  | Fully Disagree | |
| --- | --- | --- | --- | --- | --- | --- | --- | --- |
|  | 1 | | 2 | 3 | 4 | 5 | 6 | |
| In our relationship, I’m the more responsible person. |  | |  |  |  |  |  | |
| In our relationship, I’m the more caring person. |  | |  |  |  |  |  | |
| Usually, I’m the one who decides. |  | |  |  |  |  |  | |
| Usually, I find it hard to decide and leave decisions to my partner. |  | |  |  |  |  |  | |
| We make decisions together. |  | |  |  |  |  |  | |
| My partner makes the decisions outside the home and I decide at home. |  | |  |  |  |  |  | |
| I make the decisions outside the home and my partner decides at home.  I think that it’s the woman’s responsibility to care for her family and home. |  | |  |  |  |  |  | |
| I think that it’s the man’s responsibility to provide for his family. |  | |  |  |  |  |  | |
| I think that both partners should share responsibilities and domestic duties within the family. |  | |  |  |  |  |  | |
| I rather like to be alone. |  | |  |  |  |  |  | |
|  | |  | | | | | |  |

What is your religion?

| Roman Catholic | Lutheran |
| --- | --- |
| Free Evangelical | Muslim |
| Jewish | Russian Orthodox |
| Greek Orthodox | Hindu |
| None | Other: |
|  |  |

Would you describe yourself as a religious person?

| Spiritual | Somewhat religious | |  |
| --- | --- | --- | --- |
| Very religious | Not religious | |  |
| Religious |  | |  |
|  | |  | |

When you were 15 years old, what was your father’s and mother’s occupational status:

Father:

| Unskilled worker | Civil servant upper / senior service |
| --- | --- |
| White collar worker | Self-employed larger business |
| Self-employed farmer | Liberal professions, self-employed professional |
| Semi-skilled worker | Father not working |
| Manager | Homemaker |
| Skilled worker | Father deceased |
| Civil servant lower / middle service | Father unknown |
| Self-employed small / medium-sized business |  |

Mother:

| Unskilled worker | Civil servant upper / senior service | |  |
| --- | --- | --- | --- |
| White collar worker | Self-employed larger business | |  |
| Farmer | Liberal professions, self-employed professional | |  |
| Semi-skilled worker | Mother not working | |  |
| Manager | Homemaker | |  |
| Skilled worker | Mother deceased | |  |
| Civil servant lower / middle service |  | |  |
| Self-employed small / medium-sized business |  | |  |
|  | |  | |

Do you smoke or have you smoked in the past?

| No, never. | I used to, but I quit. | |  |
| --- | --- | --- | --- |
| Yes, I smoke. |  | |  |
|  | |  | |

Do you exercise?

| Yes, regularly | Occasionally |
| --- | --- |
| Yes, but not regularly | No |
|  |  |

Have you ever donated blood?

| Yes | No | |  |
| --- | --- | --- | --- |
|  | |  | |

Are you registered as a bone marrow donor?

| Yes | No | |  |
| --- | --- | --- | --- |
|  | |  | |

Do you give money to needy people on the street?

| Yes, often | Occasionally | |  |
| --- | --- | --- | --- |
| Not very often | No | |  |
|  | |  | |

In which area(s) do you volunteer in an organization, support group, sports club, or other group or project?

*Multiple answers are possible!*

| Public volunteer positions | Environment, housing |
| --- | --- |
| Church | Social work |
| Sports and exercise | Third world, human rights |
| Arts / Culture | Animal welfare |
| Political involvement and lobbying | Volunteer fire brigade, rescue service |
| School and youth | None of the above |
|  |  |

I found understanding this questionnaire…

| Very easy | Difficult | |  |
| --- | --- | --- | --- |
| Easy | Impossible | |  |
|  | |  | |

I found the topic of the questionnaire…

| Very interesting | Not very interesting |
| --- | --- |
| Interesting | Not interesting |
|  |  |

**Thank you for completing the questionnaire!**
